# Current state of evidence-based practice in clinical physiotherapy

**DOI:** 10.4102/sajp.v81i1.2139

**Published:** 2025-05-29

**Authors:** Ronel Roos

**Affiliations:** 1 Department of Physiotherapy, Faculty of Health Sciences, University of the Witwatersrand, Johannesburg, South Africa

**Keywords:** clinical practice, evidence-based practice, evidence-based physiotherapy, physiotherapy, physical therapy

## Abstract

Evidence-based practice (EBP) is a process that a clinician undertakes, implementing his or her clinical expertise while collaborating with the patient or client in the sociocultural context of care using the best available research evidence to inform practice. The approach has evolved over time in relation to its name, definition, scope and specific steps. In October 2001, member organisations of World Physiotherapy (then the World Confederation for Physical Therapy) gathered in London to discuss the status of EBP in the profession and to gain buy-in from regions to ensure that EBP would become a culture of practice in all regions. The purpose of this article is to describe the current state of EBP during physiotherapy as presented in the literature. Two decades have passed since this meeting, with noteworthy developments in clinical practice, education and research synthesis. Physiotherapists’ opinions and attitudes towards EBP are favourable but do not always ensure good implementation and execution during clinical practice. Standardised outcome measures to evaluate the EBP of physiotherapists are available. Barriers remain, which are multifactorial, and facilitators such as time, support and education are integral to optimise implementation. The launch of the Physiotherapy Evidence Database is one strategy that facilitates EBP in physiotherapy.

**Clinical implications:**

A culture of EBP exists upon reflection of published literature from different world regions. Consistent, quality implementation of EBP, including shared decision making with patients and evaluation of evidence implemented during clinical practice remain necessary.

## Introduction

Evidence-based practice (EBP) is a fundamental component of healthcare. The primary goal of EBP is to empower the clinician to make informed decisions. It has evolved over time when considering the concept’s name, definition and scope. Evidence-based practice is a movement that started in the 1990s and initially related solely to the medical profession (Djulbegovic & Guyatt 2017). In 1991, Gordon H. Guyatt introduced the term evidence-based medicine (EBM) (Guyatt 1991). The EBM Working Group, led by Gordon H. Guyatt, resided with McMaster University in Canada (Herbert 2011). It was felt that clinicians were not using research evidence during practice, relying more on their own beliefs regarding care, even when research showed that new developments could improve patients’ lives if implemented (Herbert 2011). The process outlined for EBM included sourcing research evidence, critically appraising the evidence and synthesising the information retrieved to inform patient care (Djulbegovic & Guyatt 2017; Guyatt 1991). The focus was on the importance of the research evidence, with less emphasis placed on the clinician’s intuition and clinical experience when making clinical decisions regarding practice (Guyatt et al., 1992). Because of the escalation of published research literature at that time, being efficient in finding and evaluating the literature was an essential clinical skill for clinicians to acquire to practice EBM (Guyatt 1991; Guyatt et al. 1992). A skill still crucial in today’s practice.

A change occurred, narrowing the gap between research evidence and clinical expertise in the understanding of EBM. In 1997, David Sackett highlighted that using external evidence (research literature) alone to assist with clinical decision-making was insufficient to support EBM (Sackett 1997). David Sackett was one of the founders of EBP (Moseley et al. 2020). He stressed that the clinician’s experience, expertise and, therefore, practice knowledge, in addition to research evidence and being cognisant of the patient’s concerns, values and beliefs, are necessary during clinical practice when implementing EBM (Sacket 1997). He introduced the concept of a ‘good doctor’, indicating that such a practitioner implement EBM with the above-mentioned multifaceted components (Sackett 1997).

Similarly to Sackett (1997), Kleiner et al. (2023), in a recent integrative review, highlight what constitutes good practice in physiotherapy by outlining what patients and physiotherapists alike consider a ‘good’ physiotherapist to be. The authors state that such an individual should be responsive to patients, caring, demonstrate ethical qualities, be an effective communicator and be competent. Competence is further unpacked as being knowledgeable, having clinical experience, being reflective, being confident, being curious, being able to clinically reason and having practical skills that ensure good patient outcomes (Kleiner et al. 2023). All attributes necessary to aid in following the steps of EBP and practice evidence-based physiotherapy.

The Sicilian Statement on EBP was published in 2005, following the International Conference of Evidence-Based Health Care Teachers and Developers in September 2003. This statement was constructed following a review of the literature on evidence-based healthcare and incorporating the views of conference delegates. This statement proposed a name change from EBM to EBP (Dawes et al. 2005). This name change occurred as other healthcare professions embraced the EBP approach. In addition, the benefits of the entire healthcare team following an evidence-based paradigm during clinical practice were recognised (Dawes et al. 2005). Since then, more research evidence is available outlining the benefits of EBP. In a recent scoping review of 636 articles, Connor et al. (2023) mapped the outcome of EBP programmes implemented in healthcare settings across healthcare disciplines. The authors found that such programmes can potentially improve patient outcomes, healthcare return on investments made and optimise patient satisfaction (Connor et al. 2023).

The definition of EBP as supported in the Sicilian Statement was:

Evidence-based Practice (EBP) requires that decisions about health care are based on the best available, current, valid and relevant evidence. These decisions should be made by those receiving care, informed by the tacit and explicit knowledge of those providing care, within the context of available resources. (Dawes et al. 2005:4)

**FIGURE 1:**
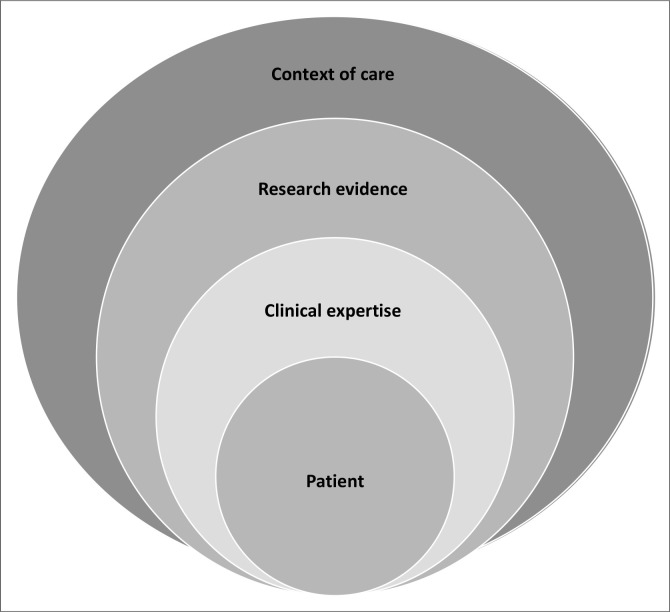
Components of evidence-based practice.

This definition of EBP embraced the earlier sentiments stated by Sackett (1997) but added the importance of the healthcare context and resource availability in the context of care as additional factors to consider when implementing EBP (see Figure 1).

In October 2001, member organisations of World Physiotherapy (then the World Confederation for Physical Therapy) gathered in London to discuss the status of EBP in the profession (Stewart 2003). Furthermore, to get buy-in from regions to ensure that EBP would become the norm and part of the specific culture of practice in all regions as we advance (Stewart 2003). Twenty years have passed since this meeting, with noteworthy developments in clinical practice, teaching and learning and research synthesis.

This commentary aims to answer the following question:

*What is EBP and its current state in clinical physiotherapy?*

Literature on the steps and process of EBP, the opinions of physiotherapists towards EBP, how physiotherapists implemented evidence-based physiotherapy during clinical practice and the known barriers and facilitators are discussed in order to make recommendations for future developments.

## Steps of evidence-based practice

Understanding the process and steps related to EBP is essential to facilitate optimal communication between parties. In addition, reviewing the steps highlights how the components of EBP – the clinician’s experience and expertise, the use of research evidence and the patient’s preferences – are integral to EBP. Literature also suggests that inefficient application of the process and steps of EBP are still apparent in healthcare today (Connor et al. 2023). Lastly, a lack of knowledge and training on EBP are barriers to EBP during physiotherapy clinical practice, as demonstrated in recent literature from Africa and the Middle East (Alshehri et al. 2017; ShahAli et al., 2023; Silumesii, Damba & Magapatona 2024).

During the early phase of EBM, which later changed to EBP, a distinct five-step process was outlined (Dawes et al. 2005; Dusin, Melanson & Mische-Lawson 2023; Herbert 2011; Hoffmann, Bennett & Del Mar 2023; Sackett 1997). Table 1 contains these five steps, which are labelled accordingly. A seven-step process was later proposed as an alternative, where step zero is cultivating a spirit of enquiry, and the last step relates to disseminating the EBP results (Melnyk et al. 2010).

**TABLE 1:** Steps that outline the process of evidence-based practice.

Step	Task	Explanation
-	*Assess* the patient.	Begin with the patient and formulate the clinical problem after conducting a comprehensive assessment.
-	*Cultivate* a spirit of enquiry.	Be curious regarding practice and develop an attitude of asking questions.
1	*Ask* the clinical question.	Create the clinical question using assessment findings as justification for the question in the PICOT format if possible.
2	*Acquire* the best evidence.	Select resources and databases, and search for the evidence.
3	*Appraise* the evidence.	Review the evidence and consider the validity and clinical applicability of the evidence sourced.
4	*Apply* the findings to practice.	Use the evidence with your clinical expertise and consider the patient’s values, beliefs and expectations to inform the decision regarding patient care to be implemented.
5	*Evaluate* the outcome.	Evaluate your performance during the process and the patient’s outcome to determine if and where your management can improve.
	Disseminate the EBP results.	Share the outcome with the broader community.

*Source*: Information in Table 1 was sourced from Sackett, D.L., 1997, ‘Evidence- based medicine’, *Seminars in Perinatology* 21(1), 3–5. https://doi.org/10.1016/S0146-0005(97)80013-4, Melnyk, B.M., Fineout-Overholt, E., Stillwell, S.B. & Williamson, K.M., 2010, ‘Evidence-based practice: step by step: The seven steps of evidence-based practice’, *American Journal of Nursing* 110(1), 51–53. https://doi.org/10.1097/01.NAJ.0000366056.06605.d2, Dusin, J., Melanson, A. & Mische-Lawson, L., 2023, ‘Evidence-based practice models and frameworks in the healthcare setting: A scoping review’, *BMJ Open* 13(5), e071188. https://doi.org/10.1136/bmjopen-2022-071188

EBP, evidence-based practice; PICOT, patient and/or population, intervention, comparator, outcomes, time.

Clinical decision-making remains complex, relying on the clinician’s ability to collect, evaluate, interpret and synthesise information from many sources. It includes clinical reasoning and problem-solving which require the clinician to use research evidence, information from patient assessment, known practice knowledge (clinician experience and expertise) and patient preferences to inform decisions (Abdu et al. 2024; Dawes et al. 2005; Herbert 2011; Sackett 1997). Thoughtful assessment, using appropriate standardised outcome measures consistently to gather evidence, and being reflective during clinical practice are components of EBP (Abdu et al. 2024). All of these aspects are integral to the steps of EBP.

The practice has moved away from the viewpoint that the clinician makes the decisions for the patient to be more inclusive and consider patient preferences (Herbert 2011; Hoffmann et al. 2023; Welding & Smith 2013; World Physiotherapy 2023). Therefore, shared decision-making is the focus, making use of clinicians’ ‘soft skills’ during practice: communication, attitude, etiquette, situational awareness, empathy, active listening, respect and building rapport with the patient, which are essential (Metgud & D’Silva 2021; Ogden et al. 2021). In addition, it includes assessment of patient-reported outcomes using patient-reported outcome measures during patient evaluation, if applicable (Weldring & Smith 2013). Therefore, placing the patient central to the process of EBP as depicted in Figure 1.

The World Physiotherapy’s definition of EBP encapsulates this newer contemporary model of practice: ‘Evidence should be integrated with clinical experience, taking into consideration beliefs, values and the cultural context of the local environment, as well as patient/client preferences’ (World Physiotherapy 2023:1). Implementation of EBP during clinical care is influenced by several factors, one of which could be the opinions of the end-user.

### Opinions of physiotherapists towards evidence-based practice

A systematic review provides information on the opinions of physiotherapists regarding EBP from seven studies conducted across the globe (Australia, Brazil, Canada, France, Germany, Sweden and the United States). Viewpoints from physiotherapists included in these studies consider the practice necessary, and reviewing research evidence to inform practice is important (Da Silva et al. 2015). Physiotherapists also believe that the availability of research evidence during practice aids their clinical decision-making and that implementing EBP has the potential to improve the quality of care provided to patients (Da Silva et al., 2015; Gleadhill et al. 2022; Mwololo et al. 2021).

The attitude of physiotherapists towards EBP seems to be positive, as reported in the literature from Australia, Colombia, India, Kenya and Saudi Arabia (Alshehri et al. 2017; Chaturvedi et al. 2021; Gleadhill et al. 2022; Mwololo et al. 2021; Ramírez-Vélez et al. 2015). Having a positive attitude towards EBP does not necessarily relate to implementing consistent and good-quality EBP during clinical physiotherapy (Scurlock-Evans, Upton & Upton 2014). Literature provides evidence on whether physiotherapists implement the steps of EBP during clinical practice and how this process changes over time.

## Evidence-based practice in physiotherapy

Considering the importance of EBP in healthcare, it is vital to assess clinicians’ implementation of EBP during clinical practice. Tools are available to allow researchers to assess this construct. Fernández-Dominquez et al. (2014) conducted a systematic review, collated the tools used in research studies to evaluate EBP in clinical physiotherapy and reviewed the reliability and validity of the respective tools. The authors identified 24 instruments in the review but concluded that they all had shortcomings in relation to the constructs included to evaluate EBP in clinical physiotherapy (Fernández-Domiquez et al. 2014). Balzer et al. (2023), in a more recent systematic review, indicated that the Evidence-Based Practice Inventory (EBPI), Evidence-Based Practice Confidence (EPIC) scale and Health Sciences-Evidence-Based Practice (HS-EBP) questionnaire are tools available with sufficient content validity, reliability and internal consistency to assess EBP in healthcare practitioners, including physiotherapy. Therefore, the ability to assess EBP during clinical physiotherapy with standardised tools has improved.

Physiotherapists’ beliefs and confidence regarding the ability to conduct EBP activities influence clinical practice implementation. Literature from an Australian study, including in part information collected with the EPIC scale, suggests that allied health professionals, for example, physiotherapists, occupational therapists, among others, with no formal postgraduate qualifications begin to lose confidence related to EBP activities within the first 5 years of clinical practice (Klaic, McDermott & Haines 2019b). This involves using research evidence to inform clinical decision-making (Klaic et al. 2019b). Specifically, activities involving critical analysis of published studies (Klaic et al. 2019b). The authors suggest that this loss of confidence may be because of early-career clinicians’ limited opportunities to practise these skills during clinical practice. In addition, there may be a lack of support and guidance from senior colleagues who have lost their confidence in conducting EBP activities (Klaic et al. 2019b). In a second qualitative study, the authors noted that the lack of practice and implementation of EBP steps during clinical practice leads to a degradation of these skills (Klaic, McDermott & Haines 2019a). This then, in turn, results in efficiencies when clinicians search for research evidence, and the process is then deemed too time-consuming (Klaic et al., 2019a). Not using research evidence during clinical practice is concerning, as this could imply that patients do not receive evidence-based care.

Condon et al. (2016), in a scoping review of 25 studies, collated evidence that evaluated whether physiotherapists can undertake the steps of EBP during clinical practice. Studies from Africa, the Middle East, the Far East and South America on the topic of interest were not available for inclusion in the review. The authors found that asking the question (step 1), acquiring the evidence (step 2) and appraising the evidence (step 3) were steps of the EBP process mostly utilised by physiotherapists (Table 1). Step 4 – applying the findings to practice and step 5 – evaluating the outcome, require improvement. This indicates that shared decision-making, including patient preferences, clinical reasoning and evaluation of the impact of EBP, still has room for improvement during clinical physiotherapy. Findings in the scoping review indicated that physiotherapists tended to prefer using colleagues as evidence sources (Condon et al. 2016). Physiotherapists believed that feedback from their colleagues was informed by their experience and, therefore, more relevant to the specific clinical question and context of practice (Condon et al. 2016). Gleadhill et al. (2022) also found that colleagues’ treatment choices and input influenced physiotherapists’ decision-making regarding EBP. The creation of networks and their influence on EBP during physiotherapy practice are evident.

**FIGURE 2:**
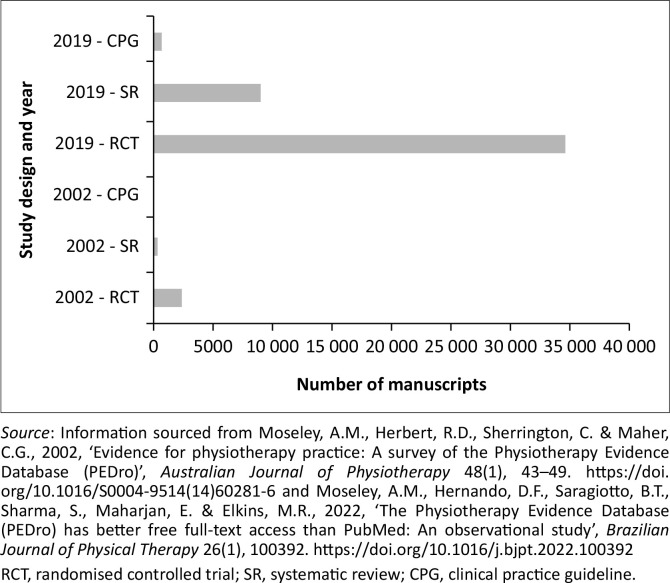
Research evidence – Randomised controlled trials, systematic reviews and clinical practice guidelines.

The creation of research evidence, for example, randomised controlled trials (RCTs) and synthesis of research evidence, for example, systematic reviews (SRs) and clinical practice guidelines (CPGs) as part of EBP and to inform evidence-based physiotherapy, has significantly increased over time (Moseley et al. 2002, 2020). In 2002, a substantial body of high-level evidence (RCTs, *n* = 2376 and SRs, *n* = 332) was available for clinicians to use to inform the process of EBP on the Physiotherapy Evidence Database (PEDro) (Moseley et al., 2002) (Figure 2). As of August 2019, 34 619 RCTs, 9004 SRs and 686 CPGs are available to clinicians (Moseley et al. 2020) (Figure 2). Disseminating the outcomes of interventions, as outlined by Melnyk et al. (2010), is a key step in the EBP process.

### Barriers to evidence-based practice

Multiple barriers, however, still exist that influence the successful implementation of EBP in clinical practice. Several reviews from across regions provide information on the barriers and facilitators of EBP in physiotherapy practice. Barriers are multifactorial and related to the individual, the work environment, the patient context and the clinician’s prior teaching and learning.

Clinicians report that a lack of time during clinical practice because of their workload and staff shortages influences the ability to engage in EBP (Paci et al. 2021; Scurlock-Evans et al., 2014; Silumesii et al. 2024). Inadequate research skills, difficulty understanding statistics in publications, and in rare instances, a lack of interest in EBP were also noted as barriers (Alshehri et al. 2017; Paci et al. 2021; Scurlock-Evans et al. 2014; Silumesii et al. 2024). In addition, physiotherapists sometimes have misconceptions regarding the process and steps of EBP (Pedersen et al. 2024; Scurlock-Evans et al. 2014).

Factors related to the work environment include inadequate support from employers and colleagues, a lack of resources and infrastructure limitations (Paci et al. 2021; Scurlock-Evans et al. 2014; ShahAli et al. 2023; Silumesii et al. 2024). Authors also reported that evidence generated with certain studies was often not generalisable to their local contexts (Paci et al. 2021; Scurlock-Evans et al. 2014; Silumesii et al. 2024). The lack of access to publications and language of publications, for example, articles mainly available in English, are additional barriers reported in studies, especially from developing countries (Paci et al. 2021; ShahAli et al. 2023). Cost of resources was also flagged as a barrier to EBP in some areas, for example, India and Africa (Chaturvedi et al., 2021; Silumesii et al. 2024).

Literature containing evidence collected with outcome measures that are not sensitive to patients’ cultural and ethnic concerns and, therefore, not relevant and applicable to the specific context of care is reported (Sawadogo et al. 2024; Silumesii et al. 2024). This demonstrates that new outcome measures sensitive to such a sociocultural context are needed or that known measures should undergo cross-cultural adaptation and validation. The general patients’ preferences and expectations may also influence how effectively EBP is implemented (Gleadhill et al. 2022; ShahAli et al. 2023).

Lastly, the literature from Africa, the Middle East and India reported a lack of knowledge of EBP or a lack of training on EBP, highlighting the need for further teaching and learning (Alshehri et al. 2017; Chaturvedi et al. 2021; ShahAli et al. 2023; Silumesii et al. 2024). The literature outlines enablers and facilitators of EBP to improve the uptake of EBP during clinical practice.

### Facilitators of evidence-based practice

Physiotherapists are more likely to be able to practice using EBP if they have sufficient time to allow for shared decision-making and increase the uptake of patient preferences in practice (Pedersen et al. 2024). The availability of standardised outcome measures and knowledge on how to use these measures improves the uptake of EBP in clinical physiotherapy as outlined in literature from Africa (Silumesii et al. 2024). The importance of training through undergraduate curricula containing content on EBP (Silumesii et al. 2024) and further postgraduate training endeavours of clinicians are strong facilitators and enablers of EBP (Klaic et al. 2019b; Silumesii et al. 2024). Klaic et al. (2019b) found that having a postgraduate qualification and more years of clinical experience influenced clinicians’ confidence in conducting activities related to EBP. How long ago postgraduate training took place did also not influence the clinicians’ confidence and subsequent ability to perform EBP over time (Klaic et al. 2019b).

Having mentorship, clinical networks, belonging to a professional group can improve clinicians’ ability to implement EBP (Condon et al. 2016; Gleadhill et al. 2022; Klaic et al. 2019a, 2019b; Silumesii et al. 2024). In addition, fostering a positive and active culture towards EBP at the workplace enhances implementation of EBP during physiotherapy (Gleadhill et al. 2022; Silumesii et al. 2024). The ability to access research evidence for evidence-based physiotherapy has greatly improved since the development and launch of the PEDro.

### Physiotherapy evidence database

The PEDro was launched in October 1999 to support EBP in physiotherapy teaching and clinical practice by providing easy access to high-level evidence, including RCTs and SRs (Moseley et al. 2002). The online resource is free and contains abstracts, as well as links to full manuscripts. The clinical trials are rated according to the PEDro scale to provide clinicians with information on the quality of the evidence published (Moseley et al. 2002). This platform has expanded exponentially and is now the most comprehensive resource for RCTs, reviews and CPGs for physiotherapy (Moseley et al., 2020). Accessing free full-text articles through PEDro is also easier compared to PubMed (Moseley et al. 2022). Articles indexed in PEDro cover all clinical physiotherapy areas and are grouped into 10 subdisciplines, of which musculoskeletal and cardiothoracic contain the most articles (Moseley et al. 2020). In celebration of its 20th anniversary in 2019, additional enhancements were initiated (Moseley et al. 2020). These enhancements included training materials already available in 13 languages were expanded to also include Arabic and Ukrainian. Content distribution platforms are now available worldwide, and the addition of subdiscipline categories was introduced. A new database, the Diagnostic Test Accuracy Database (DiTA), was also launched (Moseley et al. 2020). The DiTA (Available: https://dita.org.au/) provides information on the accuracy of diagnostic tests used by physiotherapists to further enhance EBP (Moseley et al. 2020).

### The use of large language models to inform evidence-based practice

With the advent of large language models such as ChatGPT, Claude artificial intelligence (AI) and Perplexity AI, among others, accessing information is much quicker. Literature suggests that the use of AI application models in healthcare may support human decision-making (Sharma et al. 2022). Hao et al. (2025) report that ChatGPT demonstrates promise as a supplementary decision-making tool for musculoskeletal physiotherapy, with good accuracy and reliability found when comparing responses to known clinical practice guideline recommendations. Further research is needed to refine its scope and clinical applicability (Hao et al. 2025). The use of AI could in future influence how we implement evidence-based physiotherapy during clinical practice.

## Recommendations

Literature suggests that undergraduate curricula in some regions need to be updated to include the teaching and learning of EBP to students. Evidence-based practice involves lifelong learning and the need to upskill oneself when gaps in clinical and research skills are identified. Findings from this article indicate that participation in postgraduate training and attaining additional qualifications optimises the implementation of EBP during clinical practice. The creation and participation in continual professional development, such as workshops, short courses, diplomas, or additional postgraduate degrees (MSc and PhD), create future opportunities for clinicians and academics to optimise EBP.

Over the last 20 years, strides have been made in creating outcome measures in all spheres of physiotherapy practice, for example, intensive care versus outpatients and urban versus rural. In addition, the psychometric properties of such tools are available for review to assess their applicability for practice. Regular reflective practice is needed to ensure a range of outcome measures are implemented during practice to improve the clinical execution of EBP and provide proof of the evidence of physiotherapy treatment implemented. This includes patient-reported outcomes with patient-reported outcome measures.

Further research is needed in cross-cultural adaptation and validating existing outcome measures into languages other than English to increase the utility of these tools across regions. In the absence of an existing tool relevant to a specific outcome, which is culturally sensitive within the context of care, a collaboration between clinicians and researchers is needed to create and test such a tool.

The availability of time during clinical practice, support at the workplace and resources influence the implementation of EBP. Addressing these organisational factors is warranted to optimise evidence-based patient care.

The presence of large language models, such as, ChatGPT, Claude AI and Perplexity AI, among others, are here to stay, and they have the potential to positively and negative influence education, research and clinical practice. How to implement and what is acceptable use during EBP requires discussion and further research.

## Conclusion

A culture of EBP in clinical physiotherapy is in existence upon reflection of published literature from different regions of the world, and one can therefore say that the aim of the World Physiotherapy meeting held in London in 2001 has been achieved. Patients have the right to quality, evidence-based patient care to optimise their rehabilitation. When physiotherapists undertake the steps of EBP to implement research evidence into clinical practice, it has the potential to optimise patient outcomes, impact funding of services rendered and enhance patient satisfaction through shared decision-making. Improvements can still occur with regards to the method and quality of EBP conducted, as highlighted in this article.

## References

[CIT0001] Abdu, S.I., Maikarfe, A.H., Gambo, H.B., Tanko, I.M. & Sani, F. S., 2024, ‘Chapter 2: Clinical measurement as a resource for evidence-based practice in physiotherapy’, in H. Nakano (ed.), Physical therapy towards evidence-based practice, pp. 1–13, IntechOpen, London. 10.5772/intechopen.111144

[CIT0002] Alshehri, M.A., Alalawi, A., Alhasan, H. & Stokes, E., 2017, ‘Physiotherapists’ behaviour, attitudes, awareness, knowledge and barriers in relation to evidence-based practice implementation in Saudi Arabia: A cross-sectional study’, JBI Evidence Implementation 15(3), 127–141. 10.1097/XEB.0000000000000106 PMC5592985 28399014

[CIT0003] Balzer, J., Jung, A., Gerhard, J., Reinecke, S., Mijic, M., Fichtmüller, A. et al., 2023, ‘Psychometric properties of questionnaires to assess evidence-based practice among occupational, physical and speech therapists: A systematic review’, Zeitschrift für Evidenz, Fortbildung und Qualität im Gesundheitswesen 176, 1–11. 10.1016/j.zefq.2022.11.003 36702639

[CIT0004] Chaturvedi, R., Khrolia, S., Yadav, V. & Bagri, M., 2021, ‘Knowledge, attitude and implementation of evidence-based practice of physiotherapists in India: A web-based cross-sectional study’, Romanian Journal of Neurology 20(2), 228–232. 10.37897/RJN.2021.2.16

[CIT0005] Condon, C., McGrane, N., Mockler, D. & Stokes, E., 2016, ‘Ability of physiotherapists to undertake evidence-based practice steps: A scoping review’, Physiotherapy 102(1), 10–19. 10.1016/j.physio.2015.06.003 26404896

[CIT0006] Connor, L., Dean, J., McNett, M., Tydings, D.M., Shrout, A., Gorsuch, P.F. et al., 2023, ‘Evidence-based practice improves patient outcomes and healthcare system return on investment: Findings from a scoping review’, Worldviews on Evidence-Based Nursing 20(1), 6–15. 10.1111/wvn.12621 36751881

[CIT0007] Dawes, M., Summerskill, W., Glasziou, P., Cartabellotta, A., Martin, J., Hopayian, K. et al., 2005, ‘Second international conference of evidence-based health care teachers and developers. Sicily statement on evidence-based practice’, BMC Medical Education 5(1), 1–7. 10.1186/1472-6920-5-1 15634359 PMC544887

[CIT0008] Da Silva, T.M., Costa, L.D.C.M., Garcia, A.N. & Costa, L.O.P., 2015, ‘What do physical therapists think about evidence-based practice? A systematic review’, Manual Therapy 20(3), 388–401. 25458142 10.1016/j.math.2014.10.009

[CIT0009] Djulbegovic, B. & Guyatt, G.H., 2017, ‘Progress in evidence-based medicine: A quarter century on’, Lancet 390(10092), 415–423. 10.1016/S0140-6736(16)31592-6 28215660

[CIT0010] Dusin, J., Melanson, A. & Mische-Lawson, L., 2023, ‘Evidence-based practice models and frameworks in the healthcare setting: A scoping review’, BMJ Open 13(5), e071188. 10.1136/bmjopen-2022-071188 PMC10230988 37217268

[CIT0011] Fernández-Domínguez, J.C., Sesé-Abad, A., Morales-Asencio, J.M., Oliva-Pascual-Vaca, A., Salinas-Bueno, I. & De Pedro-Gómez, J.E., 2014, ‘Validity and reliability of instruments aimed at measuring evidence-based practice in physical therapy: A systematic review of the literature’, Journal of Evaluation in Clinical Practice 20(6), 767–778. 10.1111/jep.12180 24854712

[CIT0012] Gleadhill, C., Bolsewicz, K., Davidson, S.R.E., Kamper, S.J., Tutty, A., Robson, E. et al., 2022, ‘Physiotherapists’ opinions, barriers, and enablers to providing evidence-based care: A mixed-methods study’, BMC Health Services Research 22(1), 1382. 10.1186/s12913-022-08741-5 36411428 PMC9677623

[CIT0013] Guyatt, G., Cairns, J., Churchill, D., Cook, D., Haynes, B., Hirsh, J. et al., 1992, ‘Evidence-based medicine: A new approach to teaching the practice of medicine’, Journal of the American Medical Association 268(17), 2420–2425. 10.1001/jama.1992.03490170092032 1404801

[CIT0014] Guyatt, G.H., 1991, ‘Evidence-based medicine’, American College of Physician Journal Club March/April, 114, A–16.

[CIT0015] Hao, J., Yao, Z., Tang, Y., Remis, A., Wu, K. & Yu, X., 2025, ‘Artificial intelligence in physical therapy: Evaluating ChatGPT’s role in clinical decision support for musculoskeletal care’, Annals of Biomedical Engineering 53, 1–5. 10.1007/s10439-025-03676-4 39760952

[CIT0016] Herbert, R, 2011, Chapter 1: Evidence-based Physiotherapy: What, how and why?, viewed 08 January 2025, from https://acrobat.adobe.com/id/urn:aaid:sc:EU:be2426d3-e87f-40e6-ac1e-6e462205cb40.

[CIT0017] Hoffmann, T., Bennett, S. & Del Mar, C., 2023, ‘Introduction to evidence-based practice’, in T. Hoffmann, S. Bennett & C. Del Mar (eds.), Evidence-based practice across the health professions, 4th edn., pp. 1–13, Elsevier Health Sciences, Sydney.

[CIT0018] Klaic, M., McDermott, F. & Haines, T., 2019a, ‘Does the theory of planned behaviour explain allied health professionals’ evidence-based practice behaviours? A focus group study’, Journal of Allied Health 48(1), 43E–51E. 30826841

[CIT0019] Klaic, M., McDermott, F. & Haines, T., 2019b, ‘How soon do allied health professionals lose confidence to perform EBP activities? A cross-sectional study’, Journal of Evaluation in Clinical Practice 25(4), 603–612. 10.1111/jep.13001 30178627

[CIT0020] Kleiner, M.J., Kinsella, E.A., Miciak, M., Teachman, G., McCabe, E. & Walton, D.M., 2023, ‘An integrative review of the qualities of a “good” physiotherapist’, Physiotherapy Theory & Practice 39(1), 89–116. 10.1080/09593985.2021.1999354 34881685

[CIT0021] Melnyk, B.M., Fineout-Overholt, E., Stillwell, S.B. & Williamson, K.M., 2010, ‘Evidence-based practice: Step by step: The seven steps of evidence-based practice’, American Journal of Nursing 110(1), 51–53. 10.1097/01.NAJ.0000366056.06605.d2 20032669

[CIT0022] Metgud, S. & D’Silva, P.V., 2021, ‘Soft skills in physical therapy profession: Need of the hour’, Indian Journal of Physical Therapy and Research 3(2), 67–69. 10.4103/ijptr.ijptr_77_21

[CIT0023] Moseley, A.M., Elkins, M.R., Van der Wees, P.J. & Pinheiro, M.B., 2020, ‘Using research to guide practice: The physiotherapy evidence database (PEDro)’, Brazilian Journal of Physical Therapy 24(5), 384–391. 10.1016/j.bjpt.2019.11.002 31813695 PMC7563998

[CIT0024] Moseley, A.M., Herbert, R.D., Sherrington, C. & Maher, C.G., 2002, ‘Evidence for physiotherapy practice: A survey of the Physiotherapy Evidence Database (PEDro)’, Australian Journal of Physiotherapy 48(1), 43–49. 10.1016/S0004-9514(14)60281-6 11869164

[CIT0025] Moseley, A.M., Hernando, D.F., Saragiotto, B.T., Sharma, S., Maharjan, E. & Elkins, M.R., 2022, ‘The Physiotherapy Evidence Database (PEDro) has better free full-text access than PubMed: An observational study’, Brazilian Journal of Physical Therapy 26(1), 100392. 10.1016/j.bjpt.2022.100392 35158222 PMC8850732

[CIT0026] Mwololo, T.K., Olivier, B., Karuguti, W.M. & Matheri, J.M., 2021, ‘Attitudes, perceptions and barriers around evidence-based practice in sports physiotherapy in Kenya’, South African Journal of Physiotherapy 77(1), 1561. 10.4102/sajp.v77i1.1561 34522819 PMC8424747

[CIT0027] Ogden, K., Kilpatrick, S., Elmer, S. & Rooney, K, 2021, ‘Attributes and generic competencies required of doctors: Findings from a participatory concept mapping study’, BMC Health Services Research 21(1), 560. 10.1186/s12913-021-06519-9 34098942 PMC8186188

[CIT0028] Paci, M., Faedda, G., Ugolini, A. & Pellicciari, L., 2021, ‘Barriers to evidence-based practice implementation in physiotherapy: A systematic review and meta-analysis’, International Journal of Quality Health Care 33(2), 1–13. 10.1093/intqhc/mzab093 34110410

[CIT0029] Pedersen, S.K., Platzer, O.J., Rathleff, M.S. & Hoegh, M., 2024, ‘“What scientific evidence supports this?” How do physiotherapists in private practice use evidence-based practice and what are the main challenges? A convergent parallel mixed-methods study’, European Journal of Physiotherapy 26(4), 201–210. 10.1080/21679169.2023.2234404

[CIT0030] Ramírez-Vélez, R., Correa-Bautista, J.E., Muñoz-Rodríguez, D.I., Ramírez, L., González-Ruíz, K., Domínguez-Sánchez, M.A. et al., 2015, ‘Evidence-based practice: Beliefs, attitudes, knowledge, and skills among Colombian physical therapists’, Colombia Médica 46(1), 33–40. 26019383 PMC4437285

[CIT0031] Sackett, D.L., 1997, ‘Evidence-based medicine’, Seminars in Perinatology 21(1), 3–5. 10.1016/S0146-0005(97)80013-4 9190027

[CIT0032] Sawadogo, A., Sogbossi, E.S., Everard, G.J., Kpadonou, T. & Batcho, C.S., 2024, ‘Use of standardised outcome measures among physiotherapists in French-speaking sub-Saharan Africa’, South African Journal of Physiotherapy 80(1), 1981. 10.4102/sajp.v80i1.1981 38322653 PMC10839157

[CIT0033] Scurlock-Evans, L., Upton, P. & Upton, D, 2014, ‘Evidence-based practice in physiotherapy: A systematic review of barriers, enablers and interventions’, Physiotherapy 100(3), 208–219. 10.1016/j.physio.2014.03.001 24780633

[CIT0034] ShahAli, S., Kajbafvala, M., Fetanat, S., Karshenas, F., Farshbaf, M., Hegazy, F. et al., 2023, ‘Barriers and facilitators of evidence-based physiotherapy practice in Iran: A qualitative study’, Musculoskeletal Care 21(4), 1507–1528. 10.1002/msc.1831 37818988

[CIT0035] Sharma, M., Savage, C., Nair, M., Larsson, I., Svedberg, P. & Nygren, J.M., 2022, ‘Artificial intelligence applications in health care practice: Scoping review’, Journal of medical Internet Research 24(10), e40238. 10.2196/40238 36197712 PMC9582911

[CIT0036] Silumesii, L., Damba, D. & Magapatona, A., 2024, ‘Barriers and facilitators to evidence-based practice among physiotherapists practicing in sub-Saharan Africa’, Open Journal of Therapy and Rehabilitation 12(4), 316–346. 10.4236/ojtr.2024.124024

[CIT0037] Stewart, A., 2003, ‘Evidence-based Physiotherapy’, PhysioForum, viewed 07 October 2024, from https://www.saphysio.co.za/media/178437/physioforum-february-2003.pdf.

[CIT0038] Weldring, T. & Smith, S.M.S., 2013, ‘Article commentary: Patient-reported outcomes (PROs) and patient-reported outcome measures (PROMs)’, Health Services Insights 6, HSI.S11093. 10.4137/HSI.S11093 PMC4089835 25114561

[CIT0039] World Physiotherapy, 2023, Evidence-based practice: Policy statement, viewed 30 September 2024, from https://world.physio/sites/default/files/2024-01/PS-2023-EBP.pdf.

